# Ignore fractures of the ulnar styloid in distal radius fractures

**DOI:** 10.1186/1753-6561-9-S3-A38

**Published:** 2015-05-19

**Authors:** Andrzej Zyluk

**Affiliations:** 1 Department of General and Hand Surgery, Pomeranian Medical University, Szczecin, 70-204, Poland

## Introduction

Fractures of the ulnar styloid frequently accompany fractures of the distal radius, however, there is no conclusive evidence whether it influences the final outcome of the treatment. The majority of studies show no negative effect of ulnar styloid fractures on final outcome, when the fracture of the distal radius is fixed, preferably by a locking plate [1,2]. Some authors, however, report on frequent non-unions of the ulnar styloid, distal radio-ulnar joint (DRUJ) instabilities, triangular fibrocartilage complex (TFCC) tears, and other complications which may cause ulnar wrist pain, reduced mobility and power of the hand [3,4]. No studies are available that address this issue for K-wire fixation of the distal radius fractures.

## Objective

A prospective study was carried out to compare outcomes after K-wire fixation of distal radius fractures with and without associated ulnar styloid fracture.

## Patients and methods

Seventy patients, 60 women (86%) and 10 men (14%) with a mean age of 63 years were enrolled. Thirty-five patients had isolated fracture of the distal radius and 35 had associated fracture of the ulnar styloid. All patients underwent percutaneous, “augmented” K-wire fixation of the distal radius fracture; the ulnar styloid fracture was left untreated. The patients were followed-up at 3 and 6 months. The stability of the distal radioulnar joint and the DASH scores were considered to be primary outcome measures.

## Results

### Functional assessments

At 3 months assessment there were no statistically significant differences between the groups, except for ulnar deviation, which was significantly greater in the isolated DRF group (24^O^ vs 21^O^); however, such a slight difference is not clinically meaningful. Distal radio-ulnar joint was found stable in all patients with isolated DRF and unstable in two patients with concomitant ulnar styloid fractures. These two patients had the highest DASH scores - 67 and 71, suggesting poorer function.

At 6 months assessment there were no statistically significant differences between the groups in all considered parameters. Likewise at 3 months assessment, DRUJ was found unstable in the same two patients with ulnar styloid fractures, who continued to have the highest DASH scores - 64 and 53. None of them decided to undergo surgery for this instability until the end of the study.

### Radiological assessments

Assessment at 3 months showed no statistically significant differences between the groups, except for radial inclination, which was significantly lower in the group with concomitant ulnar styloid fracture (20^O^ vs 16^O^). The number of patients with radiological parameters regarded as unacceptable was: dorsal tilt >20^O^ had two patients with isolated DRF vs one with associated ulnar styloid fracture. Ulnar variance < -5 mm had only two patients with isolated DRF and radial inclination <15^O^ had five patients in each group.

Assessment at 6 months showed no statistically significant differences between the groups in radiological parameters of the distal radius. Number of patients with radiological parameters regarded as unacceptable was: dorsal tilt >20^O^ had three patients with isolated DRF vs one with associated ulnar styloid fracture. Ulnar variance < -5 mm had only two patients with isolated DRF, and radial inclination <15^O^ had five in either group.

Ulnar styloid was found to be united in three patients (9%) at 3 months and in eight (in total) at 6 months (23%).

## Discussion

An argument supporting the role of intact ulnar styloid for stability of the DRUJ is that it provides attachment for triangular fibrocartilage complex, volar and dorsal radioulnar ligaments. Fracture of the ulnar styloid may cause disruption or stretching of these structures, subsequently impairing the DRUJ stability. Some authors question the importance of this mechanism, suggesting that the peripheral portion of the TFCC, usually injured at the fractures of the distal radius is well vascularized and has potential to heal if the ligaments are approximated [5]. It is believed that anatomical reduction and stable fixation of the distal radius provides also well reduction of ruptured TFCC which can heal without separate addressing [1]. Next, it has been suggested that the central structure responsible for stability of the DRUJ may be the interosseous membrane. The role of this structure, particularly its “distal oblique bundle” as a strong DRUJ stabilizer was emphasized by Noda et al. [6]. These authors suggest that, despite the TFCC rupture or the presence of an ulnar styloid fracture, the stability of DRUJ is maintained when the interosseous membrane is intact and the distal portion of the radius (particularly the sigmoid notch) is stable fixed [6]. These findings question the role of the ulnar styloid in maintaining DRUJ congruity and support the opinion that stable fixation of the distal radius fracture is paramount for a satisfactory outcome.

## Conclusion

An unrepaired ulnar styloid fracture does not affect the outcome of a distal radius fracture which is fixed by “augmented” K-wire method. This finding extends the treatment options for these fracture configurations which were, as yet, suggested to be fixed by volar locking plates.
